# Elimination and reemergence of measles in Mexico: historical and structural review of the 2025-2026 outbreak

**DOI:** 10.17843/rpmesp.2026.433.16250

**Published:** 2026-08-25

**Authors:** Laura Mojica-López, Oswaldo Huchim-Lara, Orlando Paredes-Ceballos

**Affiliations:** 1 Universidad Marista de Mérida, Yucatán, Mexico Universidad Marista de Mérida Universidad Marista de Mérida Yucatán Mexico; 2 Hospital Faro del Mayab, Mérida, Yucatán, Mexico Hospital Faro del Mayab Mérida Yucatán Mexico

**Keywords:** Measles, Vaccination, Mexico, Social Determinants of Health

## Abstract

A documentary analysis of historical, normative, epidemiological, and scientific sources of the structural conditions linked to measles in Mexico was performed to describe the disease's trajectory, examine vaccination coverage gaps, and understand the factors that favored its reemergence after regional elimination. Following the interruption of endemic transmission in 1995 and regional elimination in 2016, the disease reemerged in 2025-2026 in a context of accumulation of susceptible population, incomplete vaccination schedules, and territorial inequalities in access to health services. The outbreak concentrated in territories with suboptimal coverages and higher social vulnerability, which evidenced that the sustainability of elimination does not depend solely on vaccine availability, but also on the health system's capacity to maintain homogeneous coverages, sensitive epidemiological surveillance, and timely responses to case importation.

## INTRODUCTION

Measles is an exanthematous febrile disease caused by the measles virus, belonging to the family *Paramyxoviridae* and the genus *Morbillivirus*^1^. Its transmission occurs through the dissemination of airborne droplets or by direct contact with nasopharyngeal secretions from infected individuals when speaking, coughing, or sneezing ^2^. Globally, measles vaccination has been one of the most effective public health interventions, preventing approximately 60.3 million deaths between 2000 and 2023. However, the global coverage of the first dose of measles vaccine (MCV1) receded during the COVID-19 pandemic, decreasing to 81.0% in 2021 and stagnating at 83.0% during 2022 and 2023, which has favored the resurgence of outbreaks globally ^3^.

In 2016, as a result of decades of sustained vaccination strategies, epidemiological surveillance, and response to imported cases, the Region of the Americas was declared free of endemic measles transmission, becoming the first region in the world to achieve this milestone ^4^. However, the sustainability of this achievement depends on maintaining homogeneous vaccination coverage and robust epidemiological and laboratory surveillance systems ^5^^,^^6^. In the Region of the Americas, elimination was the result of coordinated efforts between countries, follow-up vaccination campaigns, active surveillance of febrile exanthematous diseases, and rapid response to imports ^6^. Even with these advances, heterogeneity in population immunity and the persistence of suboptimal coverage have generated foci of susceptibility that facilitate the reintroduction and rapid spread of the virus ^3^^,^^6^. In Mexico, endemic transmission was interrupted in 1995 and, since 1996, registered cases were classified as imported or import-associated ^4^^,^^7^.

Following the 2020 outbreak, in which 196 cases were confirmed mainly in the central region of the country, Mexico did not register any between 2021 and 2023. Subsequently, in 2024, seven import-linked cases were identified, reflecting the persistence of epidemiological vulnerability conditions ^8^. Unlike official reports, which describe the operational evolution of the outbreak, this article integrates the historical trajectory of measles in Mexico, the accumulated vaccination gaps, the regional context of elimination and reemergence, and the social determinants that conditioned population susceptibility. Therefore, the objective of this article is to describe the historical and epidemiological trajectory of measles in Mexico, analyze vaccination gaps, and examine the structural determinants that contributed to its reemergence during the 2025-2026 outbreak.

## METHODOLOGICAL APPROACH

A documentary analysis of historical, normative, epidemiological, and scientific secondary sources was conducted. Official reports from the Mexican Secretariat of Health, the Pan American Health Organization (PAHO), the World Health Organization (WHO), normative documents on vaccination and epidemiological surveillance, as well as scientific literature indexed in databases such as PubMed, SciELO, and LILACS, were consulted. The article was organized into four axes: the historical evolution of measles in Mexico, vaccination strategies and regional elimination, the recent epidemiological situation, and structural determinants related to re-emergence.

## BACKGROUND AND INTRODUCTION TO MEXICO

Measles has been one of the infectious diseases with the greatest historical impact in Mexico. Its introduction to the American continent dates back to 1495, with Juan de Aguado's expedition to Santo Domingo ^9^. At the national level, the first documented outbreak occurred in 1531, probably introduced by Spaniards who disembarked in Veracruz ^10^; indigenous peoples called this condition *tepitonzahuatl* or "small leprosy" ^9^^,^^11^. Due to the absence of previous immunity, this event had devastating demographic consequences on the indigenous population and contributed to the population collapse during the conquest period. The chronicles of the time, such as those by Fray Toribio de Benavente, recount how the disease spread "like fire" through the central valleys ^10^, particularly in the child population, consolidating itself as a health problem of great relevance ^11^.

During the 20th century, measles maintained an endemic behavior with periodic epidemics. Between 1922 and 1974, almost half a million deaths were documented, with an average mortality rate of 38.6 per 100,000 inhabitants ^7^^,^^12^. This behavior reflected the sustained circulation of the virus in a population with a high proportion of susceptibles, characteristic of the prevaccination period. The introduction of vaccination in 1970 marked the beginning of a substantial and progressive change in the epidemiological pattern of measles, with biennial epidemic outbreaks and subsequently, with wider intervals between them ^7^. The last major national epidemic episode was between 1989 and 1990, with 89,163 confirmed cases, which prompted the restructuring of national immunization strategies ^7^. As a result of these efforts, the endemic transmission of measles was interrupted in 1995 and, from 1996, registered cases were classified as imported or import-associated ^4^.

This process culminated in 2016 with the regional declaration of measles elimination in the Americas by PAHO ^4^. The elimination of endemic transmission did not imply the disappearance of risk; in a scenario of constant international mobility, the sustainability of this achievement depends on the maintenance of homogeneous coverage, of sensitive epidemiological surveillance, and of timely laboratory confirmation of suspected cases ^5^^,^^6^. In subsequent years, areas with suboptimal coverage persisted, associated with territorial and social inequalities, particularly in rural and highly marginalized contexts, where geographical barriers and access to health services hindered timely vaccination ^13^. These conditions did not immediately translate into sustained transmission, but rather favored a progressive accumulation of susceptibles in specific territories. The COVID-19 pandemic exacerbated these vulnerabilities by affecting the continuity of essential services ^14^, testing a model that had managed to interrupt endemic circulation, but which required constant surveillance to be sustained over time.

## VACCINATION

The history of measles immunization in Mexico formally began in 1970 with the local production of the Schwarz strain, consolidating in 1973 with the National Immunization Program ^15^. This effort marked the beginning of a sustained transformation in the epidemiology of the disease. In the years prior to the incorporation of new combined vaccines, significant rubella circulation persisted, with outbreaks reported in 50 municipalities in 1996, 89 in 1997, and 56 in 1998, in addition to concerns about the risk of infection during pregnancy. In this epidemiological context, the triple viral vaccine (MMR), which protects against measles, mumps, and rubella, was incorporated in 1998, and the divalent vaccine against measles and rubella (MR) was introduced in 2000. Starting in 2001, the expansion of the national schedule incorporated this vaccine into the child and adolescent population and also contemplated its application in adults, thus extending immunization against both diseases beyond the previously established childhood schedule ^(^^7^^,^^15^.

After the interruption of endemic transmission in 1995, the main challenge ceased to be the control of recurrent epidemics and shifted to focusing on the preservation of the achievement reached. Maintaining homogeneous coverages above 95.0% became a priority objective ^6^, given that, even in an elimination scenario, small territorial gaps can allow the reintroduction and spread of the virus.

Despite this, coverage was not uniformly distributed throughout the country. The dispersion of rural localities, geographical barriers, the concentration of services in urban areas, and the institutional fragmentation of the health system hindered effective access and timely follow-up of vaccination schedules. To these barriers were added educational and health literacy inequalities, which could limit the active search for vaccination and participation in school or community campaigns, particularly in municipalities with higher levels of marginalization ^13^^,^^16^.

Between 2006 and 2024, an accumulated gap of 22.5 million doses was estimated between the MMR doses required for the target population and those registered as administered. This gap occurred in a context of persistent programmatic difficulties, characterized by inconsistencies between vaccine acquisition and administration, variability among institutions and years, and discrepancies between estimated and reported coverages. These limitations, added to the territorial and access barriers previously described, could have favored lost vaccination opportunities and the progressive accumulation of susceptible population ^16^. In this scenario, misinformation and vaccine hesitancy should be considered additional contextual factors that can reduce timely vaccination demand, especially when combined with low institutional trust or exposure to unverified messages ^17^^,^^18^. However, it is not possible to attribute the decline in coverages in Mexico mainly to organized anti-vaccine movements. Nevertheless, in the Region of the Americas, PAHO has warned that vaccine misinformation can affect the perception of safety and efficacy of biologicals, so addressing it is part of the necessary strategies to sustain vaccine acceptance and prevent new outbreaks ^17^.

During the COVID-19 pandemic, immunization programs faced logistical adjustments and a reduction in both demand and access. Worldwide, first-dose coverage was 83.0%, and heterogeneous decreases were recorded in Mexico, with border municipalities and marginalized communities presenting coverages below 80.0% ^3^^,^^16^. The 2022 National Health and Nutrition Survey (ENSANUT) estimated a 61.8% application of the first dose of MMR ^19^, a figure that evidenced a significant gap compared to the threshold necessary to sustain collective immunity. Between 2022 and 2024, adjustments were made to the national vaccination schedule. The first dose of MMR remained at 12 months, and the second dose was advanced from 6 years to 18 months, in order to favor early protection; for those born before 2021, the booster at 6 years was maintained ^15^. These decisions sought to close accumulated gaps and reduce windows of susceptibility in early childhood.

The effectiveness of the vaccine applied in Mexico has remained high, estimated at 98.1% during the recent health crisis ^13^. Therefore, the reemergence of the disease observed in subsequent years should not be attributed to a decrease in the vaccine's effectiveness, but to the persistence of incomplete vaccination schedules and the progressive accumulation of susceptible population in subnational foci, particularly in Chihuahua, where historically low coverages and long-standing vaccination gaps were documented. In this way, the vaccination program, although solid in its regulatory design, faced difficulties in sustaining homogeneous coverages in a scenario characterized by social inequality and high population mobility within and outside the country.

In this scenario, average national coverages could generate a perception of stability that did not reflect the existing territorial heterogeneity. The accumulation of susceptible individuals was not uniform, but rather localized, allowing chains of transmission to be established in specific territories following the introduction of the virus.

## RECENT EPIDEMIOLOGICAL SITUATION (2024-2026)

After the outbreak recorded in 2020, in which 196 cases were confirmed mainly in the central part of the country ^20^, Mexico went through a period of epidemiological silence between 2021 and 2023 ^8^.

This interval was consistent with the regional elimination scenario in effect until that time, but it did not negate the risk of reintroduction. In 2024, seven import-related cases were identified; the first corresponded to a 4-year-old child, originally from Romania, detected in March in Mexico City after arriving from London ^8^^,^^14^. These events showed that, even in the absence of sustained transmission, the risk of reintroduction remained latent.

From February 2025, a sustained increase in confirmed cases was documented, constituting the most significant outbreak observed in the country since the interruption of endemic transmission ^13^. Between 2025 and 2026, a heterogeneous territorial distribution was recorded, with a higher concentration of cases in Jalisco, Chihuahua, Chiapas, and Mexico City ^21^. This pattern evidenced a focalized transmission in specific federal entities, although with the presence of cases in most of the national territory. The geographical distribution of accumulated confirmed cases by federal entity is shown in Figure 1.

**Figure 1:**
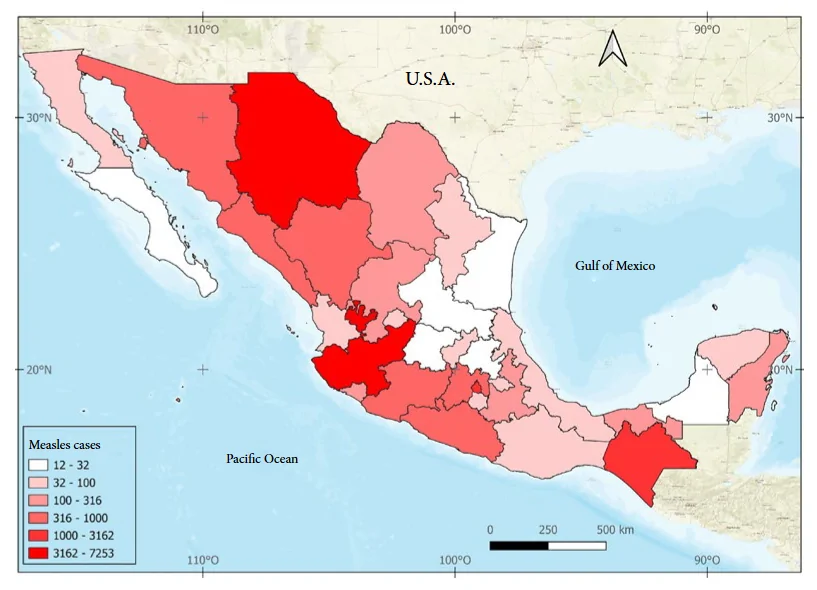
Geographical distribution of accumulated confirmed cases of measles by federal entity, Mexico, 2025-2026.

Source: Own elaboration with data from the Secretariat of Health/General Directorate of Epidemiology, daily report of the measles outbreak in Mexico, June 22, 2026.

As of epidemiological week 42 of 2025, 91.7% of confirmed cases had no vaccination history, which reflected the previous accumulation of a susceptible population in certain territories ^22^. This behavior was not nationally homogeneous, but was concentrated in specific areas where coverage had been insufficient or irregular in previous years.

Molecular analysis identified two viral genotypes; B3 was detected only in Oaxaca, and D8 (Ontario.CAN lineage), which represented 98.1% of the analyzed sequences ^13^. The predominance of genotype D8 confirmed the introduction of the virus through international transmission chains. The outbreak dynamics followed three stages: an introduction associated with labor mobility, an amplification in urban communities with low acceptance or access to vaccination, and a subsequent spread to marginalized indigenous populations or those with greater structural barriers to care ^13^, where clinical and epidemiological control is more complex.

Although the most affected age group was 1 to 4 years old, with 1,327 cases, infants under 1 year old presented the highest incidence rate in the country, 50.41 per 100,000 inhabitants ^23^. The indigenous population represented three out of ten cases, but accounted for 76.0% of deaths ^13^. This distribution reflects a differentiated vulnerability, associated both with incomplete vaccination schedules and the impossibility of having sufficient protection in the first months of life. Likewise, the severity of the impact was not uniform and was influenced by structural conditions that limit timely access to vaccination, diagnosis, and medical care, especially in rural and highly marginalized contexts.

The magnitude of the event, as well as the evidence of sustained transmission in Mexico, was part of a regional reemergence context. In November 2025, PAHO notified the loss of measles elimination status in the Region of the Americas ^24^. Thus, the outbreak not only constituted a national epidemiological event but also had regional repercussions, demonstrating health interdependence in contexts of high international mobility.

The situation observed in Mexico was part of a regional phenomenon of measles reemergence in the Americas. Although the region was the first in the world to achieve elimination in 2016, this advance had already faced setbacks due to sustained outbreaks in Venezuela and Brazil, with temporary loss of certification and subsequent recovery in 2024 through strengthened surveillance, follow-up vaccination campaigns, and regional cooperation ^4^^,^^6^^,^^24^. During 2025, a new increase in cases was observed in several countries; by November, close to 12,600 confirmed cases had been reported in ten countries, with an approximate concentration of 95.0% in Canada, Mexico, and the United States ^24^. This resurgence occurred in a context of insufficient coverages, including a regional second-dose coverage of close to 79.0% in 2024, below the 95.0% recommended to interrupt transmission ^24^. Together, the regional experience demonstrates that sustaining elimination requires high and homogeneous coverages, sensitive epidemiological and laboratory surveillance, and timely responses to new cases.

## IMPLICATIONS FOR THE SUSTAINABILITY OF ELIMINATION

The reemergence of measles in Mexico between 2025 and 2026 should not be interpreted as an isolated event, but as the manifestation of accumulated structural vulnerabilities that persisted even after the interruption of endemic transmission in 1995 and the regional declaration of elimination in 2016 ^4^^,^^8^. Although elimination steadily reduced incidence, it did not eliminate the need to maintain homogeneous coverages greater than 95.0% nor to sustain sensitive and timely epidemiological surveillance systems ^6^.

The geographical concentration of the outbreak in specific territories evidenced that population immunity was not uniform ^13^. National averages could suggest stability; even so, the existence of territorial gaps allowed the reintroduction of the virus to find active transmission chains. The progressive accumulation of susceptible individuals in certain communities constituted a silent process that only became visible when the virus was reintroduced ^(^^16^.

The observed sequence (introduction associated with international mobility, amplification in communities with low coverage, and subsequent impact on indigenous populations) ^13^, reflects the interaction between epidemiological factors and social determinants. The greater burden of disease and lethality in contexts of marginalization exposed that vulnerability is not homogeneously distributed, but rather based on structural inequalities that condition timely access to vaccination, diagnosis, and medical care.

The COVID-19 pandemic exacerbated these gaps by affecting the continuity of vaccination services ^6^, generating documented declines in coverage at global and national levels ^3^^,^^19^. Although the effectiveness of the biologic used in Mexico remained high ^13^, collective protection depends less on its intrinsic efficacy than on the system's ability to ensure timely and homogeneous compliance with the vaccination schedule.

The loss of regional elimination status showed that the sustainability of the program does not depend solely on the existence of a robust regulatory framework or adequate average coverage ^(^^24^, but also on the implementation of interventions focused on historically marginalized populations and territories, with priority on reducing structural inequalities.

## CONCLUSIONS

The re-emergence of measles in Mexico was not an isolated event, but the visible manifestation of territorial gaps in vaccine coverage and a progressive accumulation of susceptible individuals in a context of structural inequality. The interruption of endemic transmission and the regional declaration of elimination demonstrated that sustained control is possible; however, its permanence depends on the maintenance of homogeneous coverages, sensitive epidemiological surveillance, and a timely response to case importation.

The 2025-2026 outbreak demonstrated that elimination does not constitute a permanent condition, but a dynamic state that requires continuous efforts. Recovering and sustaining elimination status will require not only increasing vaccine coverage, but also strengthening the anticipated response capacity of the health system and focusing interventions in territories and populations with greater lags. In this regard, the sustainability of elimination depends both on constant surveillance and on health equity.
