# Supplementary material for: Elimination and reemergence of measles in Mexico: historical and structural review of the 2025-2026 outbreak
Source: Rev Peru Med Exp Salud Publica. 2026 Aug 25;43(3):384–9. doi: 10.17843/rpmesp.2026.433.16250 (PMC13718564; doi:10.17843/rpmesp.2026.433.16250)
Supplement: Translation

# Translation: Eliminación y reemergencia del sarampión en México: revisión histórica y estructural del brote 2025-2026

**Authors:** Laura Mojica-López, Oswaldo Huchim-Lara, Orlando Paredes-Ceballos

**Affiliations:** 1 Universidad Marista de Mérida, Yucatán, Mexico; 2 Hospital Faro del Mayab, Mérida, Yucatán, Mexico

**Keywords:** Sarampión, Vacunación, México, Determinantes Sociales de la Salud

## Abstract

Se realizó un análisis documental de fuentes históricas, normativas, epidemiológicas y científicas de las condiciones estructurales vinculadas al sarampión en México para describir la trayectoria de la enfermedad, examinar las brechas de cobertura vacunal y comprender los factores que favorecieron su reemergencia tras la eliminación regional. Tras la interrupción de la transmisión endémica en 1995 y la eliminación regional en 2016, la enfermedad resurgió en 2025-2026 en un contexto de acumulación de población susceptible, esquemas incompletos de vacunación y desigualdades territoriales en el acceso a los servicios de salud. El brote se concentró en territorios con coberturas subóptimas y de mayor vulnerabilidad social, lo que evidenció que la sostenibilidad de la eliminación no depende únicamente de la disponibilidad de vacunas, sino también de la capacidad del sistema de salud para mantener coberturas homogéneas, vigilancia epidemiológica sensible y respuestas oportunas ante la importación de casos.

## INTRODUCCIÓN

El sarampión es una enfermedad febril exantemática causada por el virus del sarampión, perteneciente a la familia *Paramyxoviridae* y al género *Morbillivirus*^1^. Su transmisión ocurre por la diseminación de microgotas suspendidas en el aire o por contacto directo con secreciones nasofaríngeas de personas infectadas al hablar, toser o estornudar ^2^. A nivel mundial, la vacunación contra el sarampión ha sido una de las intervenciones de salud pública más efectivas, logrando prevenir aproximadamente 60,3 millones de muertes entre los años 2000 y 2023. Sin embargo, la cobertura global de la primera dosis de la vacuna contra el sarampión (MCV1) retrocedió durante la pandemia de COVID-19, disminuyó a 81,0% en 2021 y se estancó en 83,0% durante 2022 y 2023, lo que ha favorecido el resurgimiento de brotes a nivel global ^3^.

En 2016, como resultado de décadas de estrategias sostenidas de vacunación, vigilancia epidemiológica y respuesta ante casos importados, la Región de las Américas fue declarada libre de transmisión endémica del sarampión, convirtiéndose en la primera región del mundo en alcanzar este hito ^4^. No obstante, la sostenibilidad de este logro depende del mantenimiento de coberturas de vacunación homogéneas y de sistemas de vigilancia epidemiológica y laboratorial robustos ^5^^,^^6^. En la Región de las Américas, la eliminación fue resultado de esfuerzos coordinados entre países, campañas de vacunación de seguimiento, vigilancia activa de enfermedades febriles exantemáticas y respuesta rápida ante importaciones ^6^. Aun con estos avances, la heterogeneidad en la inmunidad poblacional y la persistencia de coberturas subóptimas han generado focos de susceptibilidad que facilitan la reintroducción y rápida propagación del virus ^3^^,^^6^. En México, la transmisión endémica fue interrumpida en 1995 y, desde 1996, los casos registrados fueron clasificados como importados o asociados a importación ^4^^,^^7^.

Tras el brote de 2020, en el que se confirmaron 196 casos principalmente en la zona centro del país, México no registró ninguno entre 2021 y 2023. Posteriormente, en 2024 se identificaron siete casos vinculados a importación, lo que reflejó la persistencia de condiciones de vulnerabilidad epidemiológica ^8^. A diferencia de los informes oficiales, que describen la evolución operativa del brote, este artículo integra la trayectoria histórica del sarampión en México, las brechas acumuladas de vacunación, el contexto regional de eliminación y reemergencia, y los determinantes sociales que condicionaron la susceptibilidad poblacional. Por ello, el objetivo de este artículo es describir la trayectoria histórica y epidemiológica del sarampión en México, analizar las brechas de vacunación y examinar los determinantes estructurales que contribuyeron a su reemergencia durante el brote de 2025-2026.

## ENFOQUE METODOLÓGICO

Se realizó un análisis documental de fuentes secundarias históricas, normativas, epidemiológicas y científicas. Se consultaron informes oficiales de la Secretaría de Salud de México, Organización Panamericana de la Salud (OPS), Organización Mundial de la Salud (OMS), documentos normativos de vacunación y vigilancia epidemiológica, así como literatura científica indexada en bases como PubMed, SciELO y LILACS. El artículo se organizó en cuatro ejes: evolución histórica del sarampión en México, estrategias de vacunación y eliminación regional, situación epidemiológica reciente y determinantes estructurales relacionados con la reemergencia.

## ANTECEDENTES E INTRODUCCIÓN A MÉXICO

El sarampión ha sido una de las enfermedades infecciosas de mayor impacto histórico en México. Su introducción al continente americano se remonta a 1495, con la expedición de Juan de Aguado a Santo Domingo ^9^. En el ámbito nacional, el primer brote documentado ocurrió en 1531, probablemente introducido por españoles que desembarcaron en Veracruz ^10^; los pueblos indígenas denominaron a esta afección *tepitonzahuatl* o «pequeña lepra» ^9^^,^^11^. Debido a la ausencia de inmunidad previa, este evento tuvo consecuencias demográficas devastadoras en la población indígena y contribuyó al colapso poblacional durante el periodo de la conquista. Las crónicas de la época, como las de Fray Toribio de Benavente, relatan cómo la enfermedad se extendió «como fuego» por los valles centrales ^10^, particularmente en la población infantil, consolidándose como un problema sanitario de gran relevancia ^11^.

Durante el siglo XX, el sarampión mantuvo un comportamiento endémico con epidemias periódicas. Entre 1922 y 1974 se documentaron casi medio millón de defunciones, con una tasa de mortalidad promedio de 38,6 por cada 100 000 habitantes ^7^^,^^12^. Este comportamiento reflejaba la circulación sostenida del virus en una población con elevada proporción de susceptibles, característica del periodo prevacunal. La introducción de la vacunación en 1970 marcó el inicio de un cambio sustancial y progresivo en el patrón epidemiológico del sarampión, con brotes epidémicos bienales y posteriormente, con intervalos más amplios entre ellos ^7^. El último gran episodio epidémico nacional fue entre 1989 y 1990, con 89 163 casos confirmados, lo que impulsó la reestructuración de las estrategias nacionales de inmunización ^7^. Como resultado de estos esfuerzos, la transmisión endémica del sarampión fue interrumpida en 1995 y, a partir de 1996 los casos registrados se clasificaron como importados o asociados a importación ^4^.

Este proceso culminó en 2016 con la declaratoria regional de eliminación del sarampión en las Américas por parte de la OPS ^4^. La eliminación de la transmisión endémica no implicó la desaparición del riesgo; en un escenario de movilidad internacional constante, la sostenibilidad de este logro depende del mantenimiento de coberturas homogéneas, de la vigilancia epidemiológica sensible y de la confirmación laboratorial oportuna de los casos sospechosos ^5^^,^^6^. En los años posteriores persistieron zonas con coberturas subóptimas asociadas a desigualdades territoriales y sociales, particularmente en contextos rurales y de alta marginación, donde las barreras geográficas y de acceso a los servicios de salud dificultaron la vacunación oportuna ^13^. Estas condiciones no se tradujeron inmediatamente en una transmisión sostenida, sino que favorecieron una acumulación progresiva de susceptibles en determinados territorios. La pandemia de COVID-19 agudizó estas vulnerabilidades al afectar la continuidad de los servicios esenciales ^14^, poniendo a prueba un modelo que había logrado interrumpir la circulación endémica, pero que requería vigilancia constante para sostenerse en el tiempo.

## VACUNACIÓN

La historia de la inmunización contra el sarampión en México inició formalmente en 1970 con la producción local de la cepa Schwarz, consolidándose en 1973 con el Programa Nacional de Inmunizaciones ^15^. Este esfuerzo marcó el comienzo de una transformación sostenida en la epidemiología de la enfermedad. En los años previos a la incorporación de nuevas vacunas combinadas persistió una circulación importante de rubéola, con brotes notificados en 50 municipios en 1996, 89 en 1997 y 56 en 1998, además de la preocupación por el riesgo de infección durante el embarazo. En este contexto epidemiológico, en 1998 se incorporó la vacuna triple viral, que protege contra sarampión, rubéola y parotiditis (SRP), y en 2000 se introdujo la vacuna divalente contra sarampión y rubéola (SR). A partir de 2001, la ampliación del esquema nacional incorporó esta vacuna en la población infantil y adolescente y contempló también su aplicación en adultos, extendiendo así la inmunización contra ambas enfermedades más allá del esquema infantil previamente establecido ^7^^,^^15^.

Tras la interrupción de la transmisión endémica en 1995, el desafío principal dejó de ser el control de epidemias recurrentes y pasó a centrarse en la conservación del logro alcanzado. Mantener coberturas homogéneas superiores al 95,0% se convirtió en un objetivo prioritario ^6^, dado que, incluso en un escenario de eliminación, pequeñas brechas territoriales pueden permitir la reintroducción y propagación del virus.

Pese a ello, la cobertura no se distribuyó de forma uniforme en el país. La dispersión de localidades rurales, las barreras geográficas, la concentración de servicios en zonas urbanas y la fragmentación institucional del sistema de salud dificultaron el acceso efectivo y el seguimiento oportuno de los esquemas de vacunación. A estas barreras se sumaron desigualdades educativas y de alfabetización en salud, que pudieron limitar la búsqueda activa de vacunación y la participación en campañas escolares o comunitarias, particularmente en municipios con mayores niveles de marginación ^13^^,^^16^.

Entre 2006 y 2024 se estimó una brecha acumulada de 22,5 millones entre las dosis de SRP requeridas para la población objetivo y las registradas como aplicadas. Esta brecha se produjo en un contexto de dificultades programáticas persistentes, caracterizadas por inconsistencias entre la adquisición y aplicación de vacunas, variabilidad entre instituciones y años, y discrepancias entre las coberturas estimadas y las reportadas. Estas limitaciones, sumadas a las barreras territoriales y de acceso previamente descritas, pudieron favorecer oportunidades perdidas de vacunación y la acumulación progresiva de población susceptible ^16^. En este escenario, la desinformación y la reticencia vacunal deben considerarse factores contextuales adicionales que pueden reducir la demanda oportuna de vacunación, especialmente cuando se combinan con baja confianza institucional o exposición a mensajes no verificados ^17^^,^^18^. Sin embargo, no es posible atribuir la caída de las coberturas en México principalmente a movimientos antivacunas organizados. No obstante, en la Región de las Américas, la OPS ha advertido que la desinformación sobre vacunas puede afectar la percepción de seguridad y eficacia de los biológicos, por lo que su abordaje forma parte de las estrategias necesarias para sostener la aceptación vacunal y prevenir nuevos brotes ^17^.

Durante la pandemia por COVID-19, los programas de inmunización enfrentaron ajustes logísticos y una reducción tanto en la demanda como en el acceso. A nivel mundial, la cobertura de la primera dosis fue de 83,0%, y en México se registraron descensos heterogéneos, con municipios fronterizos y comunidades marginadas que presentaron coberturas inferiores al 80,0% ^3^^,^^16^. La Encuesta Nacional de Salud y Nutrición (ENSANUT) 2022 estimó una aplicación de la primera dosis de SRP del 61,8% ^19^, cifra que evidenció una brecha importante respecto del umbral necesario para sostener la inmunidad colectiva.

Entre 2022 y 2024 se realizaron ajustes al esquema nacional de vacunación. La primera dosis de SRP se mantuvo a los 12 meses y la segunda dosis se adelantó de los 6 años a los 18 meses, con el fin de favorecer la protección temprana; para los nacidos antes de 2021 se mantuvo el refuerzo a los 6 años ^15^. Estas decisiones buscaron cerrar brechas acumuladas y reducir ventanas de susceptibilidad en la primera infancia.

La efectividad de la vacuna que se aplica en México se ha mantenido elevada, estimada en un 98,1% durante la reciente crisis sanitaria ^13^. Por ello, la reemergencia de la enfermedad observada en los años siguientes no debería atribuirse a una disminución de la efectividad del biológico, sino a la persistencia de esquemas incompletos y a la acumulación progresiva de población susceptible en focos subnacionales, particularmente en Chihuahua, donde se documentaron coberturas históricamente bajas y brechas de vacunación de larga data. De esta manera, el programa de vacunación, aunque sólido en su diseño normativo, enfrentó dificultades para sostener coberturas homogéneas en un escenario caracterizado por desigualdad social y alta movilidad poblacional dentro y fuera del país.

En este escenario, las coberturas promedio nacionales pudieron generar una percepción de estabilidad que no reflejaba la heterogeneidad territorial existente. La acumulación de personas susceptibles no fue uniforme, sino focalizada, permitiendo que, tras la introducción del virus, se establecieran cadenas de transmisión en territorios específicos.

## SITUACIÓN EPIDEMIOLÓGICA RECIENTE (2024-2026)

Después del brote registrado en 2020, en el que se confirmaron 196 casos principalmente en la zona centro del país ^20^, México atravesó un periodo de silencio epidemiológico entre 2021 y 2023 ^8^.

Este intervalo fue consistente con el escenario de eliminación regional vigente hasta ese momento, pero no anuló el riesgo de reintroducción. En 2024 se identificaron siete casos vinculados a importación, el primero correspondió a un niño de 4 años, originario de Rumania, detectado en marzo en la Ciudad de México tras arribar de Londres ^8^^,^^14^. Estos eventos evidenciaron que, aún en ausencia de transmisión sostenida, el riesgo de reintroducción permanecía latente.

A partir de febrero de 2025 se documentó un incremento sostenido de casos confirmados, configurando el brote más importante observado en el país desde la interrupción de la transmisión endémica ^13^. Entre 2025 y 2026 se registró una distribución territorial heterogénea, con mayor concentración de casos en Jalisco, Chihuahua, Chiapas y Ciudad de México ^21^. Este patrón evidenció una transmisión focalizada en determinadas entidades federativas, aunque con presencia de casos en la mayor parte del territorio nacional. La distribución geográfica de los casos confirmados acumulados por entidad federativa se muestra en la Figura 1.

**Figura 1:** Distribución geográfica de casos confirmados acumulados de sarampión por entidad federativa, México, 2025-2026

Fuente: elaboración propia con datos de la Secretaría de Salud/Dirección General de Epidemiología, informe diario del brote de sarampión en México, 22 de junio de 2026.

Al corte de la semana epidemiológica 42 de 2025, el 91,7% de los casos confirmados no contaba con antecedente de vacunación, lo que reflejó la acumulación previa de población susceptible en determinados territorios ^22^. Este comportamiento no fue homogéneo a nivel nacional, sino que se concentró en áreas específicas donde la cobertura había sido insuficiente o irregular en años anteriores.

El análisis molecular identificó dos genotipos virales; el B3 se detectó únicamente en Oaxaca, y el D8 (linaje Ontario.CAN), que representó el 98,1% de las secuencias analizadas ^13^. La predominancia del genotipo D8 confirmó la introducción del virus a través de cadenas de transmisión internacional. La dinámica del brote siguió tres etapas, una introducción asociada a movilidad laboral, una amplificación en comunidades urbanas con baja aceptación o acceso a vacunación, y una posterior difusión hacia poblaciones indígenas marginadas o con mayores barreras estructurales de atención ^13^, donde el control clínico y epidemiológico resulta más complejo. 

Aunque el grupo etario más afectado fue el de 1 a 4 años, con 1 327 casos, los menores de 1 año presentaron la tasa de incidencia más elevada del país, 50,41 por cada 100 000 habitantes ^23^. La población indígena representó tres de cada diez casos, pero concentró el 76,0% de las defunciones ^13^. Esta distribución refleja una vulnerabilidad diferenciada, asociada tanto a esquemas incompletos de vacunación como a la imposibilidad de contar aún con protección suficiente en los primeros meses de vida. Asimismo, la gravedad del impacto no fue uniforme y estuvo influida por condiciones estructurales que limitan el acceso oportuno a la vacunación, al diagnóstico y a la atención médica, especialmente en contextos rurales y de alta marginación.

La magnitud del evento, así como la evidencia de transmisión sostenida en México, se inscribió en un contexto regional de reemergencia. En noviembre de 2025, la OPS notificó la pérdida del estatus de eliminación del sarampión en la Región de las Américas ^24^. De este modo, el brote no solo constituyó un evento epidemiológico nacional, sino que tuvo repercusiones regionales, evidenciando la interdependencia sanitaria en contextos de alta movilidad internacional.

La situación observada en México formó parte de un fenómeno regional de reemergencia del sarampión en las Américas. Aunque la región fue la primera del mundo en lograr la eliminación en 2016, este avance ya había enfrentado retrocesos por brotes sostenidos en Venezuela y Brasil, con pérdida temporal de la certificación y recuperación posterior en 2024 mediante el fortalecimiento de la vigilancia, las campañas de vacunación de seguimiento y la cooperación regional ^4^^,^^6^^,^^24^. Durante 2025 se observó un nuevo incremento de casos en varios países; hasta noviembre se habían notificado cerca de 12 600 casos confirmados en diez países, con concentración aproximada del 95,0% en Canadá, México y Estados Unidos ^24^. Este resurgimiento ocurrió en un contexto de coberturas insuficientes, incluida una cobertura regional de segunda dosis cercana al 79,0% en 2024, por debajo del 95,0% recomendado para interrumpir la transmisión ^24^. En conjunto, la experiencia regional demuestra que sostener la eliminación requiere coberturas elevadas y homogéneas, vigilancia epidemiológica y laboratorial sensible, y respuestas oportunas ante nuevos casos.

## IMPLICACIONES PARA LA SOSTENIBILIDAD DE LA ELIMINACIÓN

La reemergencia del sarampión en México entre 2025 y 2026 no debe interpretarse como un evento aislado, sino como la manifestación de vulnerabilidades estructurales acumuladas que persistieron incluso después de la interrupción de la transmisión endémica en 1995 y la declaratoria regional de eliminación en 2016 ^4^^,^^8^. Si bien, la eliminación redujo de forma sostenida la incidencia, no eliminó la necesidad de mantener coberturas homogéneas superiores al 95,0% ni de sostener sistemas de vigilancia epidemiológica sensibles y oportunos ^6^.

La concentración geográfica del brote en territorios específicos evidenció que la inmunidad poblacional no era uniforme ^13^. Los promedios nacionales podían sugerir estabilidad; aun así, la existencia de brechas territoriales permitió que la reintroducción del virus encontrara cadenas de transmisión activas. La acumulación progresiva de personas susceptibles en determinadas comunidades constituyó un proceso silencioso que solo se hizo visible cuando el virus fue reintroducido ^(^^16^.

La secuencia observada (introducción asociada a movilidad internacional, amplificación en comunidades con baja cobertura y posterior impacto en poblaciones indígenas) ^13^, refleja la interacción entre factores epidemiológicos y determinantes sociales. La mayor carga de enfermedad y letalidad en contextos de marginación expuso que la vulnerabilidad no se distribuye de manera homogénea, sino en función de desigualdades estructurales que condicionan el acceso oportuno a la vacunación, al diagnóstico y a la atención médica.

La pandemia por COVID-19 agudizó estas brechas al afectar la continuidad de los servicios de vacunación ^6^, generando descensos documentados en la cobertura a nivel global y nacional ^3^^,^^19^. Aunque la efectividad del biológico utilizado en México se mantuvo elevada ^13^, la protección colectiva depende menos de su eficacia intrínseca que de la capacidad del sistema para asegurar el cumplimiento oportuno y homogéneo del esquema de vacunación.

La pérdida del estatus regional de eliminación demostró que la sostenibilidad del programa no depende únicamente de la existencia de un esquema normativamente robusto o de coberturas promedio adecuadas ^(^^24^, sino también de la implementación de intervenciones focalizadas en poblaciones y territorios históricamente marginados, con prioridad en la reducción de desigualdades estructurales.

## CONCLUSIONES

La reemergencia del sarampión en México no fue un evento aislado, sino la manifestación visible de brechas territoriales en la cobertura vacunal y de una acumulación progresiva de personas susceptibles en un contexto de desigualdad estructural. La interrupción de la transmisión endémica y la declaratoria regional de eliminación demostraron que el control sostenido es posible; sin embargo, su permanencia depende del mantenimiento de coberturas homogéneas, de una vigilancia epidemiológica sensible y de una respuesta oportuna ante la importación de casos.

El brote de 2025-2026 demostró que la eliminación no constituye una condición permanente, sino un estado dinámico que requiere esfuerzos continuos. Recuperar y sostener el estatus de eliminación requerirá no solo incrementar la cobertura vacunal, sino también fortalecer la capacidad anticipada de respuesta del sistema de salud y focalizar intervenciones en los territorios y poblaciones con mayores rezagos. En este sentido, la sostenibilidad de la eliminación depende tanto de la vigilancia constante como de la equidad sanitaria.
